# Addressing issues of experimental design, ecological realism and local adaptation for applications of ectotherm upper thermal limits

**DOI:** 10.1242/jeb.250748

**Published:** 2025-12-02

**Authors:** Josephine C. Iacarella, Richard Chea, David A. Patterson

**Affiliations:** ^1^ Fisheries and Oceans Canada, Cultus Lake Laboratory, 4222 Columbia Valley Hwy, Cultus Lake, BC, Canada, V2R 5B6; ^2^ Fisheries and Oceans Canada, Pacific Science Enterprise Centre, 4160 Marine Dr., West Vancouver, BC, Canada, V7V 1H2; ^3^ Fisheries and Oceans Canada, Aquatic Research Cooperative Institute, School of Resource and Environmental Management, Simon Fraser University, 8888 University Dr., Burnaby, BC, Canada, V5A 1S6

**Keywords:** Critical thermal maxima, Climate change, Coho salmon, Local adaptation, Physiological plasticity

## Abstract

Upper thermal limits of ectotherms are widely used to understand and predict species' thermal responses and sensitivity to warming. These limits are often defined for species using experiments with rapid ramping temperatures that test critical thermal maxima (CT_max_). However, there are issues that arise with relying on these experimental results including (1) the influence of experimental design on thermal maxima, (2) the lack of ecological realism and (3) the potential for population-level local adaptation of upper thermal limits. We addressed these issues by comparing the CT_max_ approach with an ecologically realistic design using slower incremental temperature ramping with diel fluctuations (ITD_max_) and by applying both to evaluate local adaptation of juvenile coho salmon (*Oncorhynchus kisutch*). We compared populations from thermal regimes spanning 7° latitude and coastal to inland systems by testing three populations, combining results with a fourth population from a prior ITD_max_ study, and comparing with other studies that used CT_max_ experiments to test thermal maxima of juvenile coho salmon. Most notably, we found that unlike CT_max_ experiments, ITD_max_ results were not influenced by acclimation temperature. This stemmed from acclimation during the ITD_max_ trials, likely representing more ecologically relevant responses to longer term warming. Furthermore, local adaptation of thermal maxima, as measured by both CT_max_ and ITD_max_, was not evident for juvenile coho salmon, with no influence of population across the nine included in the cross-study examination. The results suggest the ability to use ITD_max_-based upper thermal limits across species' extents and with differing prior environmental exposure, providing a more accurate representation of responses and sensitivity to long-term warming.

## Teaser

**Highlighted Article:** Local adaptation of juvenile coho salmon and effects of prior temperature exposure are not evident when upper thermal limits are assessed using ecologically relevant thermal ramping and diel fluctuations.

## INTRODUCTION

Upper thermal limits of ectotherms are a central focus of efforts to understand and predict how species will respond to warming temperatures. They are relied on for a range of climate change-related assessments including global evaluations of species' warming sensitivity and biogeographic patterns in thermal limits (Deutsch et al., 2008; Sunday et al., 2014, 2019; Gunderson and Stillman, 2015; Bennett et al., 2018, 2019; Nati et al., 2021); regional projections of future temperatures that exceed thermal thresholds (Sinervo et al., 2010; Wallace et al., 2015; Seixas et al., 2018; Iacarella et al., 2024); and intraspecific comparisons of local adaptation (Myrick and Cech, 2000; Fangue et al., 2006; Chen et al., 2013; Zillig et al., 2023). However, the application of upper thermal limits for such purposes has raised three major issues: (1) the strong influence of experimental design on thermal limits; (2) the ecological realism, or lack thereof, of experimental designs and relevance to environmental conditions; and (3) the degree of population-level local adaptation of upper thermal limits and applicability of a single species-level value (Chown et al., 2009; Rezende et al., 2011, 2014; Santos et al., 2011; Kingsolver and Umbanhowar, 2018; Bennett et al., 2019; Desforges et al., 2023). When unaddressed, these issues can lead to potential misapplication of upper thermal limits and subsequent misinterpretation of biogeographic patterns, climate change vulnerability and physiological adaptation. Evaluations of how populations and species may respond to climate change could be improved with further development of experimental approaches and exploration into upper thermal limit plasticity.

Interpreting thermal limits as single endpoints, as well as drawing conclusions from combining values across different experimental designs, is challenged by the significant influence of experimental design on results (Rezende et al., 2011, 2014; Santos et al., 2011; Kingsolver and Umbanhowar, 2018). In general, upper thermal limits of ectotherms show asymptotic increases with (1) higher acclimation (i.e. starting) temperatures and (2) faster ramping rates in dynamic temperature experiments, and decrease down to fully tolerated temperatures with (3) duration of exposure in static temperature experiments (Terblanche et al., 2007; Rezende et al., 2011, 2014; Kingsolver and Umbanhowar, 2018; Kovacevic et al., 2019). Application of thermal limits without considering these effects may result in misleading conclusions regarding sensitivity to warming in natural environments and when making comparisons across populations and species tested in different studies. For instance, differences in thermal limits of terrestrial ectotherms across latitudes were only detected when assay duration was included as a covariate (Rezende et al., 2014). Each of the three experimental factors (acclimation, ramp rates, duration) were also mediating covariates in models of global patterns of ectotherm thermal limits across biomes (Sunday et al., 2019). Evaluating thermal limits as single threshold values can further lead to incorrect conclusions of warming sensitivity as these factors can change thermal limits by several degrees (Rezende et al., 2014; Kingsolver and Umbanhowar, 2018).

There is continued disagreement on experimental design and the importance of ecological realism when assessing upper thermal limits. Most experimental designs currently use the critical thermal maxima (CT_max_) methodology that applies rapid ramp rates of generally 18°C h^−1^ until physiological failure is observed (Becker and Genoway, 1979). Rapid ramp rates are selected to avoid potential acclimation or heat hardening and other physiological effects during longer trials, though it is acknowledged that short-term experiments may not represent thermal tolerance at longer time scales (Rezende et al., 2011, 2014; Bates and Morley, 2020). Fast temperature ramping is also known to overshoot true upper thermal limits owing to the lag time between observed loss of equilibrium (LOE) and increasing temperatures (Beitinger et al., 2000; Desforges et al., 2023). These concerns may be less critical when evaluating differences in thermal limits among populations or species as long as differences in experimental design are accounted for. However, ecological realism and the confounding effects of experimental design are important considerations when using thermal limits to understand potential warming sensitivity and exposure risk to high environmental temperatures. Organismal responses to longer-term warming representative of potential environmental temperature exposure, including diel variation, are likely more realistic (provided other needs are met such as supplying food rather than starving) and may not warrant controlling or avoiding if the goal is to understand thermal limits relevant to climate change trends in natural settings (Morash et al., 2018; Iacarella et al., 2024). Experiments using slower ramp rates have shown reduced influence of starting acclimation temperatures on upper thermal limits, likely owing to acclimation over the course of the experiments (Åsheim et al., 2020; Iacarella et al., 2024). When the confounding effect of acclimation temperature is removed, upper thermal limits can be more widely applied as endpoints across species’ distributions that are often characterized by differing environmental exposure (Iacarella et al., 2024); however, further testing of different ecologically relevant starting temperatures and ramp rates is needed to understand the full scope of potential responses under climate warming, most notably rapid extreme heat events. In addition, physiological mechanisms of metabolism, growth and stress responses differ in organisms exposed to diel variation versus static conditions (Morash et al., 2018, 2021). Though few experimental studies to date incorporate natural diel variation, these more ecologically relevant physiological responses are important for understanding phenotypic selection and physiological adaptation in the environment (Morash et al., 2018, 2021; Kraskura et al., 2024).

Another major consideration of how thermal limits are used to understand species’ climate change responses is the degree of plasticity in thermal limits, as exemplified by the effects of experimental design, and the potential for intraspecific variation through local adaptation (Bennett et al., 2019; McKenzie et al., 2021; Nati et al., 2021). Many studies apply species-level thermal limits which assumes limited effects of local adaptation (Bennett et al., 2019; Sunday et al., 2019). One global analysis on intraspecific variation in CT_max_ found relatively high intraspecific variation in CT_max_ for temperate freshwater fish compared with tropical freshwater fish and marine fish, though the many factors that could cause variation were not disentangled (e.g. body size, life history stage, standardized acclimation temperature) (Nati et al., 2021). They also found evidence of narrowing upper thermal limit plasticity with higher species’ CT_max_ (Nati et al., 2021), which was further confirmed by reduced plasticity to acclimation temperatures in CT_max_ experiments when individuals were selected for higher upper thermal limits across generations (Morgan et al., 2020). As such, adaptive capacity of upper thermal limits is known to have a ceiling, whereby warm-adapted individuals have reduced plasticity to further acclimate (Bennett et al., 2019; Morgan et al., 2020; McKenzie et al., 2021; Nati et al., 2021; Ern et al., 2023). Local adaptation to higher temperatures therefore implies less scope for additional adaptation, or increased sensitivity, to further warming at the population level, particularly when conditions are already close to upper thermal limits. However, this does not negate that warm adaptation can confer a species-level benefit for persistence. Intraspecific variation in upper thermal limits can reduce a species' sensitivity to warming through portfolio effects (i.e. variation reduces overall risk of species’ instability) or sampling effects (i.e. variation increases the likelihood of a high thermal tolerance) (McKenzie et al., 2021). Experiments comparing intraspecific upper thermal limits while controlling for confounding factors (e.g. prior exposure, body size, life stage), though limited to date, are a useful tool for evaluating the degree of intraspecific variation from local adaptation and whether applying species-level thermal limits across geographic ranges is sufficient or needs to be population specific (McKenzie et al., 2021).

We sought to examine and advance understanding of the three issues of experimental design, ecological realism and local adaptation on upper thermal limit results. We evaluated upper thermal limits as critical thermal maxima from the traditional CT_max_ design alongside a newly developed incremental thermal diel approach (ITD_max_) that applies daytime heating rates and diel fluctuations that are relevant to local environmental conditions (Iacarella et al., 2024). Using both experimental designs, we compared thermal maxima across three populations of juvenile coho salmon (*Oncorhynchus kisutch*) from BC, Canada (spanning 5° latitude). We further compared our results with a fourth BC population from previous ITD_max_ experiments (Iacarella et al., 2024) and with other published studies measuring thermal maxima of juvenile coho salmon using CT_max_ designs, encompassing a range of thermal regimes that span a minimum of 7° latitude and coastal to inland systems (Becker and Genoway, 1979; McGeer et al., 1991; Konecki et al., 1995). We tested the hypotheses that (1) ITD_max_ thermal maxima would be lower than CT_max_ results, (2) acclimation temperatures would have a greater effect on CT_max_ results than on ITD_max_ results and (3) populations from warmer watersheds, typically associated with lower latitudes, would have higher thermal maxima than those from cooler watersheds. We predicted that the difference between populations would be more apparent in CT_max_ experiments than in ITD_max_ experiments owing to slower exposure rates and increased time to acclimate in ITD_max_. Our results have important implications for experimental design and applying species-level upper thermal limits to evaluate climate change risk and adaptation.

## MATERIALS AND METHODS

**Fig. 1.**
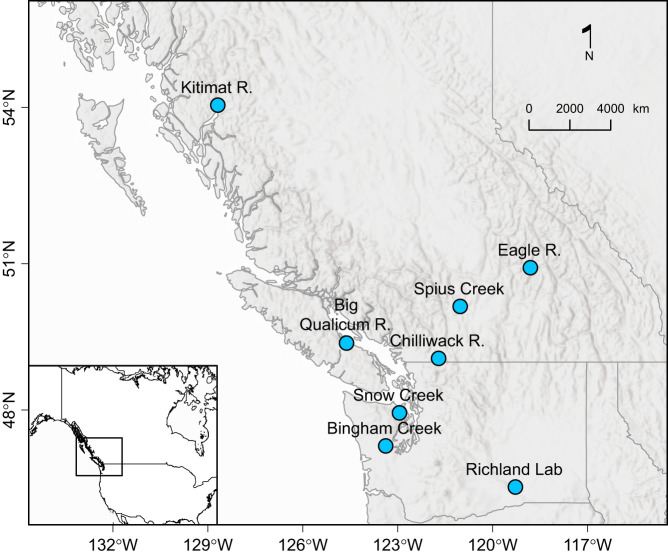
**Coho salmon population locations.** Juveniles were obtained for the current experiments from Big Qualicum River, Chilliwack River and Kitimat River, BC, Canada; and for previous thermal maxima experiments from Spius Creek and Eagle River, BC, Canada; Snow Creek and Bingham Creek, WA, USA; and an unknown population tested at Pacific Northwest Laboratories in Richland, WA, USA.

All work was conducted in compliance with Canadian Council on Animal Care Guidelines overseen by the Fisheries and Oceans Canada Pacific Region Animal Care Committee, Animal Use Protocol 24-006. Genetically distinct populations of juvenile coho salmon, *Oncorhynchus kisutch* (Walbaum 1792), were obtained from Big Qualicum River Hatchery (BQ), Chilliwack River Hatchery (CR) and Kitimat River Hatchery (KR), BC, Canada, in April 2024 (Xuereb et al., 2022) (Fig. 1). Hatcheries collected gametes from wild-caught parents and reared eggs to fry overwinter at temperatures less than 8°C. Following a minimum of 11 days of holding after transportation, fish were distributed into 195 l experimental tanks (60 per tank) filled to 50 l. Two replicate tanks were provided for each treatment (three populations, two acclimation temperatures and two experimental designs, *n*=24 tanks). All tanks were supplied with flow-through, aerated well water at a baseline temperature of 11°C. Temperature was manipulated with a series of tank heaters controlled with Remote Interface Units and a central control unit Logic Controller (InWater Technologies). Fish were held at the baseline well water temperature of 11°C for 2 weeks to reduce stress and then transitioned to acclimation temperatures (14°C and 18°C) at a rate of 1°C day^−1^. We selected 14°C and 18°C as ecologically reasonable starting temperatures for a major warming event that could lead to temperatures reaching upper thermal limits, though our cross-study comparison included acclimation temperatures down to 5°C. We used diel fluctuations throughout acclimation and trials for the ITD_max_ experiments and constant conditions (static in acclimation and ramping in trials) in CT_max_ experiments in line with the CT_max_ experimental approach (Becker and Genoway, 1979; Iacarella et al., 2024). The resulting differences between experiments therefore do not distinguish effects of thermal regime from the acclimation and experimental periods, but our focus here was a comparison of an ecologically realistic design versus the widely used CT_max_ design. For the ITD_max_ experimental tanks, diel fluctuations were introduced by increasing temperature starting at 09:00 h and then decreasing by 3°C starting at 18:00 h, followed by a 4°C increase the next day to achieve a net 1°C increase per day (Iacarella et al., 2024). Fish were acclimated for 4 weeks once both target temperatures were reached. At the end of acclimation and prior to trials, fish were size selected to remove effects of different body sizes on upper thermal limit results, retaining 51 per tank (fork length and wet mass: BQ: 5.5±0.5 cm, 2.2±0.6 g; CR: 5.5±0.3 cm, 2.1±0.4 g; KR: 5.7±0.5 cm, 2.4±0.7 g; mean±1 s.d.; *n*=8). Fish were fed to satiation using a mixture of shaved frozen mysis shrimp and bloodworms three times daily during the holding period, and feeding frequency was reduced to a single feeding 4 days a week during acclimation and throughout ITD_max_ trials. For CT_max_ experiments, fish were fasted 24 h prior to trials following standard CT_max_ methods (Becker and Genoway, 1979). Fish had a robust body condition (represented by a Fulton's *K* close to 1, using fork length and wet mass) throughout holding and trials, with a mean (±s.d.) of 1.2±0.1 across fish and sampling dates (sample dates *n*=5, total fish sampled *n*=625).

Following acclimation, water temperature in ITD_max_ experimental tanks was increased by 0.3°C day^−1^, while continuing the 3°C reduction in temperature at night, until LOE was observed for all fish (*n*=35 per tank). This temperature ramping and diel variability was selected as ecologically representative of salmon stream systems in BC (Iacarella et al., 2024). LOE was determined when fish were unable to maintain an upright position within the water column for more than 30 s. Tanks were monitored daily for signs of LOE precursors including darkening of pigmentation, surfacing, irritability, skittering, erratic or ceased swimming, long periods of inactivity, rapid respiration at the water surface or reduced appetite. Once these precursors were observed (more than 20 days after ramping started and approximately 4–5 days prior to the start of LOE), daytime monitoring was increased to hourly and then to every 15 min during the periods of highest LOE. During LOE periods, monitoring duration was extended from 12 h to up to 14 h or until no more LOE was observed for 30 min. The vast majority of fish lost equilibrium during the more heavily monitored periods and were euthanized immediately (315 out of 420 or 75%); the remaining fish were reported as overnight mortalities.

CT_max_ experiments (15–19 July 2024) were conducted after LOE of 18°C ITD_max_ fish (6–10 July 2024) and before LOE of 14°C ITD_max_ fish (20–23 July 2024) to approximate similar holding durations. Three trials were conducted daily for 4 days, alternating the order of treatments and replicate tanks tested (Table S1). Fish used in the trials (*n*=35 for each of two replicate tanks per population and acclimation temperature) were taken from the CT_max_ acclimation tanks, transferred to a testing tank (50 l) and held for 30 min prior to starting temperature ramping. Temperature was then increased 0.3°C min^−1^ following standard ramping rates (Becker and Genoway, 1979) by pumping in heated well water until LOE was observed for all fish. Upon LOE in both CT_max_ and ITD_max_ experiments, individuals were removed and immediately euthanized using MS-222 buffered with sodium bicarbonate (Morrison et al., 2020). In some CT_max_ studies, recovery is observed before euthanizing to ensure a measure of non-lethality (Zillig et al., 2023); in other cases, fish are recovered when they are re-used for additional experimentation, but then immediately euthanized following LOE in final trials (Åsheim et al., 2020; Bartlett et al., 2022). Here, we followed methods originally employed by Becker and Genoway (1979), who did not test for recovery, with the understanding that LOE is an ecological death where fish would be unable to escape heat exposure and be vulnerable to predation (Åsheim et al., 2020; Vallin et al., 2025). All fish were measured (length) and weighed following euthanasia.

### Data analysis

Thermal maxima were determined as the maximum temperature experienced before fish lost equilibrium. This was the temperature reached at the time of LOE for CT_max_ experiments, and the highest daytime temperature prior to LOE for ITD_max_ experiments (i.e. the temperature reached the previous afternoon when LOE was observed at night or in the morning). Differences in thermal maxima of juvenile coho salmon between experimental designs (ITD_max_ versus CT_max_), acclimation treatments (14 and 18°C) and populations (BQ, CR, KR) were tested using linear regression. The global model included a three-way interaction between experimental design, acclimation treatment and population, and an additive effect of final body mass to account for any potentially remaining effect of body size. The best-fit model was determined based on a ΔAICc≥10 and a significant difference between the top ranked model and the next model using ‘dredge’ (MuMIn package in R; https://cran.r-project.org/web/packages/MuMIn/index.html) and ANOVA, respectively. Significant differences between treatment levels were calculated using Tukey's honestly significant difference (HSD) tests.

Experimental results were further compared with previous thermal maxima studies on juvenile coho salmon. First, we compared ITD_max_ results with a past ITD_max_ experiment using juvenile coho salmon from Spius Creek, BC, Canada (Fig. 1). In this experiment, 15 coho salmon per treatment and replicate were acclimated to 12, 14 and 17°C, and trials applied a 1°C day^−1^ ramp rate and a 2°C night-time decline in temperature (Iacarella et al., 2024). A linear regression and Tukey's HSD were used to test differences in thermal maxima across acclimation treatments (12, 14, 17, 18°C) and populations (BQ, CR, KR, SC) from the two experiments. The effect of ramp rate or study ID was not included as this was confounded with population from the Iacarella et al. (2024) experiments (i.e. the SC population was tested under a different ramp rate to the other populations). For these four populations, temperature profiles were collated from hydrometric stations (Water Survey of Canada) near locations of the hatcheries and associated populations to approximate the thermal regimes they originate from. BQ and SC regions showed overall highest stream temperatures, and temperatures between CR and KR were lower and relatively similar (Fig. S1). For instance, August 2024 mean and maximum temperatures were 19.1±1.5°C and 21.7°C for BQ (Millstone River at Nanaimo), 13.7±1.8°C and 18.3°C for CR (Slesse Creek near Vedder Crossing), 14.8±1.7°C and 18.3°C for KR (Hirsch Creek near the mouth), and 18.2±3.1°C and 25.5°C for SC (Spius Creek near Canford), respectively.

Next, we compared our results with the three other published thermal maxima experiments on juvenile coho salmon that used a ramping temperature experimental design (Table 1). These studies included constant ramp rates ranging from 1 to 60°C h^−1^, acclimation treatments of 5–15°C and five populations (Becker and Genoway, 1979; McGeer et al., 1991; Konecki et al., 1995) (Table 1). The ramp rate from ITD_max_ experiments was calculated as the temperature increase across a 7 h window of increasing temperature based on the diel fluctuations (applying an artificial 24 h window did not change the results). We used a linear mixed effects model to test fixed effects of population, ramp rate and an interaction between experimental design and acclimation treatment, with a random effect of study ID (*n*=5) (lme4 package in R; Bates et al., 2015). Best-fit models were determined as described above.

**Table 1.** Thermal maxima studies on juvenile coho salmon populations from WA, USA and BC, Canada, using ramping temperature experimental designs

Study	Population	Experiment	Ramp rate – common units	Ramp rate – standardized units (°C min^−1^)	Acclimation (°C)
Becker and Genoway, 1979	Unknown; lab based in Richland, WA	CT_max_	1, 6, 18, 30, 60°C h^−1^	0.017, 0.1, 0.3, 0.5, 1	5, 15
McGeer et al., 1991	Chilliwack River & Eagle River, BC	CT_max_	1°C h^−1^	0.017	6
Konecki et al., 1995	Bingham Creek & Snow Creek, WA	CT_max_	18°C h^−1^	0.3	11
Iacarella et al., 2024	Spius Creek, BC	ITD_max_	1°C day^−1^ (diel)	0.002 (diel)	12, 14, 17 (diel)
Current study	Big Qualicum, Chilliwack River, & Kitimat River, BC	CT_max_	18°C h^−1^	0.3	14, 18
Current study	Big Qualicum, Chilliwack River, & Kitimat River, BC	ITD_max_	0.3°C day^−1^ (diel)	0.0007 (diel)	14, 18 (diel)

CT­_max_­, critical thermal maximum; ITD_max_, incremental thermal diel maximum.

## RESULTS

**Fig. 2.**
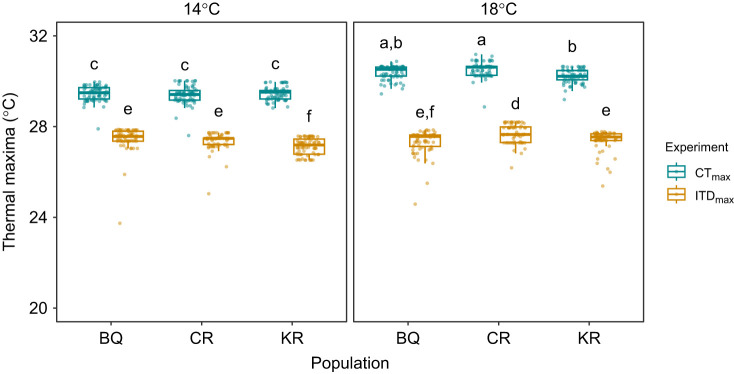
**Maximum temperature reached by juvenile coho salmon populations before loss of equilibrium (LOE) using two experimental methods.** Salmon (*n*=35) were acclimated to 14 and 18°C and exposed to rapidly increasing temperatures (critical thermal maximum, CT_max_: 0.3°C min^−1^) versus incremental and diel-fluctuating temperatures (incremental thermal diel maximum, ITD_max_: 0.3°C day^−1^, 3°C night-time decline). Different letters indicate significant differences (*P*<0.05) for the three-way interaction between experimental design, acclimation and population (BQ, Big Qualicum River; CR, Chilliwack River; KR, Kitimat River).

The best-fit model comparing experimental designs, acclimation treatments and populations from the experiments herein included a significant three-way interaction (best-fit model and next best-fit model, ΔAICc=15; three-way interaction term: *F*_2,829_=10.0, *P*<0.001). Body mass did not have an effect on thermal maxima (*P*>0.05) as there was *a priori* size selection that limited size variation across all treatments. Thermal maxima in CT_max_ experiments were 2–3°C higher than those in ITD_max_ experiments (overall CT_max_=29.9±0.6°C, ITD_max_=27.4±0.5°C, mean±1 s.d.; Fig. 2). Across populations, mean thermal maxima increased by 1°C from 14 to 18°C (29.4±0.3°C to 30.4±.03°C, respectively) in CT_max_ experiments, but only had a mean increase of 0.2°C (27.3±0.5°C to 27.5±0.5°C, respectively) in ITD_max_ experiments. Thermal maxima between populations resulted in some significant differences (*P*<0.05) that were minimal in terms of absolute temperature differences, inconsistent across treatments and not aligned with thermal regimes from the areas where they originated (Fig. S1). For instance, the greatest mean difference (0.4°C) was between BQ and CR in ITD_max_ 18°C treatments, with thermal maxima of 27.3±0.5°C and 27.7±0.4°C, respectively, but the difference was reduced (0.1°C) and reversed in 14°C treatments, with thermal maxima of 27.5±0.5°C and 27.4±0.4°C, respectively (Fig. 2).

**Fig. 3.**
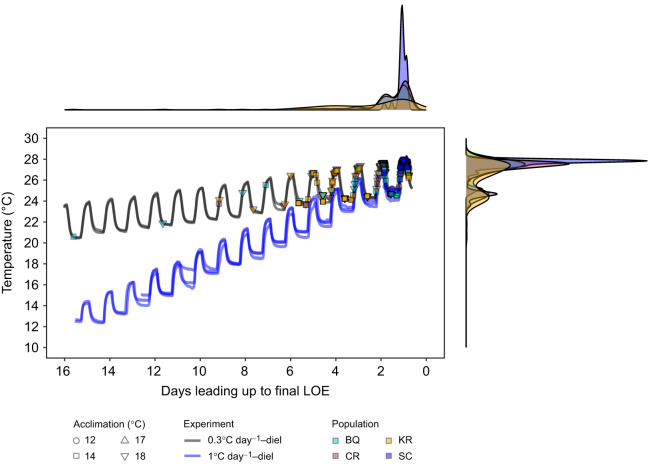
**Comparison of thermal maxima for coho salmon from slower and more rapid ITD_max_ experiments.** Aligned temperature profiles based on days leading up to the final LOE for comparison across treatments and ITD_max_ experiments presented here (black lines) and in Iacarella et al. (2024) (blue lines), and associated juvenile coho salmon LOE events. Density plots are shown for LOE events along temperature and days from endpoint axes.

**Fig. 4.**
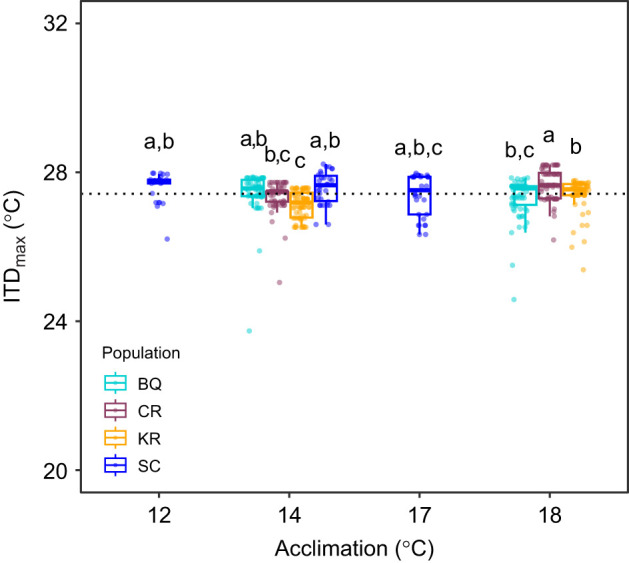
**Combined thermal maxima across four acclimation temperatures and populations from ITD_max_ experiments.** Data are from the present study and Iacarella et al. (2024). The dotted line indicates the mean thermal maximum (27.4°C) across all treatments. Different letters indicate significant differences (*P*<0.05) for the interaction between acclimation and population (BQ, Big Qualicum River; CR, Chilliwack River; KR, Kitimat River; SC, Spius Creek).

Comparison between these ITD­_max_ experiments and the faster ramping ITD_max_ experiment in Iacarella et al. (2024) revealed more variation in the timing of LOE between individuals with slower ramping temperatures, but similar endpoints overall (Fig. 3). Juvenile coho salmon in ITD_max_ trials lost equilibrium over the course of 16 and 12 days in 14°C (days 34–49 from start of temperature ramping) and 18°C treatments (days 24–35), respectively, with earliest LOE observed for BQ in both treatments (Fig. 3; Fig. S2). Conversely, LOE was observed over the course of 4 days with faster ITD_max_ ramping (Iacarella et al., 2024) (Fig. 3). In the current ITD_max_ trials, LOE occurred most frequently during daytime temperature peaks (15:00–19:00 h) and overnight (19:00–09:00 h), with continued loss the following day before the next daytime high (09:00–15:00 h) (Fig. S3). The statistical comparison of thermal maxima from the two ITD_max_ experiments resulted in a significant interaction between acclimation treatment and population (best-fit model and next best-fit model, ΔAICc=18; interaction term: *F*_2,501_=11.3, *P*<0.001). However, thermal maxima were relatively similar with no clear patterns despite some significant differences (*P*<0.05) (Fig. 4). The overall mean was 27.4±0.5°C.

**Fig. 5.**
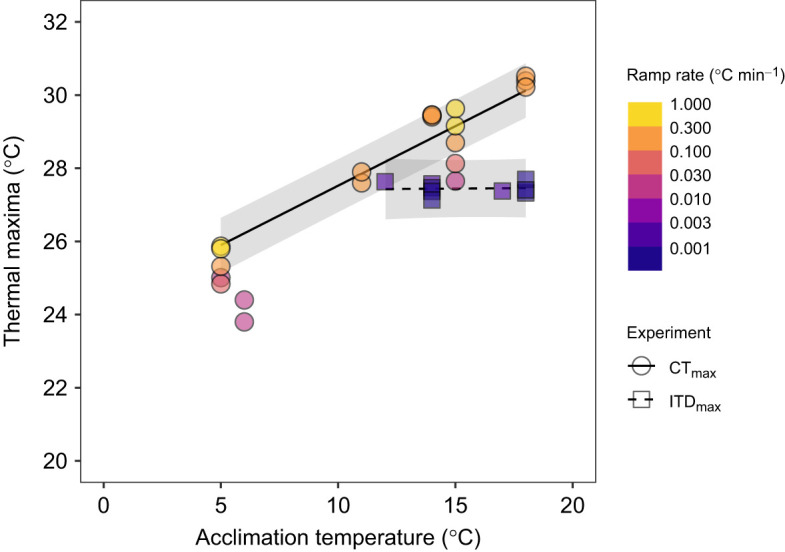
**Partial plot showing the interactive effect between acclimation temperature and experimental design on juvenile coho salmon thermal maxima from five studies (including the present one).** Points are mean thermal maxima for each study treatment and colors indicate the rate of temperature increase on a log scale. The regression lines are conditioned on a ramp rate of 0.5°C min^−1^ and shaded areas are 95% confidence bands.

Comparing thermal maxima results of experiments from the present study and other thermal maxima studies [i.e. using constant ramp rates (range 1–60°C h^−1^) on different coho populations] resulted in a best-fit model (variance explained by fixed and random effects, *r*^2^=0.98) that included a significant interaction between experimental design and acclimation treatment (estimate=0.3, s.e.=0.04, *t*=7.5; model comparison without term: χ^2^=30.4, *P*<0.001) and an additive effect of ramp rate (estimate=1.5, s.e.=0.2, *t*=6.7; model comparison without term: χ^2^=26.1, *P*<0.001) (best-fit model and next best-fit model, ΔAICc=23); the effect of population was not significant (*P*>0.05). The most notable contrast was the resulting thermal maxima slope for ITD_max_ experimental designs of 0.0 across acclimation temperatures compared with 0.3 for CT_max_ experimental designs (Fig. 5). Increasing ramp rate also increased thermal maxima as indicated in the CT_max_ experimental design results (Fig. 5).

## DISCUSSION

Our results from upper thermal limit experiments have implications for experimental design and population-level local adaptation that diverge from traditional CT_max_ studies. Most notably, slow ramping temperatures with diel fluctuations in ITD_max_ experiments led to negligible differences in thermal maxima across acclimation treatments, whereas CT_max_ experiments herein and from others yielded expected increases in thermal maxima with acclimation temperature. This indicates juvenile coho salmon were acclimating over the course of the ITD_max_ experiments such that the influence of starting temperature was diminished. The findings that (1) starting temperature (or prior exposure in the environment) had minimal importance when more realistic warming temperature profiles were used and (2) the onset of LOE among individuals was prolonged during slow temperature increases with diel reprieves provide evidence that the ITD_max_ experiments captured warming responses more relevant to seasonal and long-term temperature shifts in the environment than CT_max_ experiments. Interestingly, no meaningful biological differences or patterns were found between thermal maxima of juvenile coho salmon using either experimental design, nor was there any significant effect of nine populations across thermal regimes spanning a minimum of 7° latitude and coastal to inland systems. In this case, local adaptation of upper thermal limits was not supported. These combined results indicate ITD_max_ for coho salmon is a more ecologically relevant measure of upper thermal limits than CT_max_ for understanding responses to warming in most natural environments, and should force us to re-examine assertions of the importance of local adaptation acting on upper thermal limits for all species and life stages (Bennett et al., 2019; McKenzie et al., 2021; Mayer et al., 2024).

Rapid ramping CT_max_ experiments yielded higher thermal maxima than slow ramping, diel-fluctuating ITD_max_ experiments. The degree of acclimation that was evident in the ITD_max_ experiments from the reduced effect of different starting temperatures (i.e. 0.7–1.1°C increase from 14 to 18°C in CT_max_ experiments down to increases/decreases of 0.2–0.3°C in ITD_max_) was not sufficient to overcome the effect of ramp rates between experiments. This is likely the result of two mechanisms. First, CT_max_ experiments can overshoot true upper thermal limits owing to the rapidity of increasing temperatures and the lag time between the observation of LOE (Beitinger et al., 2000; Desforges et al., 2023). Second, thermal stress was likely accumulating over the course of the ITD_max_ experiments, which limited the extent of acclimation, similar to the negative effect of exposure duration on upper thermal limits in static temperature experiments (Rezende et al., 2014). Both of these mechanisms are supported by the finding that many individuals in the ITD_max_ experiments showed lag effects of heat stress by losing equilibrium after daytime high temperatures had subsided, and even in some cases the next morning after night-time decreases. In a related experiment on zebrafish (*Danio rerio*), thermal maxima were found to be higher using slow ramp rates (0.025°C min^−1^) than fast ramp rates (0.3°C min^−1^) when acclimated at lower temperatures, and the reverse was true at higher acclimation temperatures (Åsheim et al., 2020). It was suggested that the former case was either from acclimation during the slow ramping trials or heat hardening from exposure of the same individuals to the fast ramping trials before the slow ramping trials (Åsheim et al., 2020). Our results indicate that short-term resistance to temperature, as tested by rapid ramp rates (Bates and Morley, 2020), appears to exceed effects of acclimation at slow ramp rates. We suggest that future work examines the physiological mechanisms that are mediating thermal tolerance limits, and how they may differ between rapid and slow warming rates (Ern et al., 2023; Vallin et al., 2025).

The relevance of CT_max_ experiments versus more ecologically realistic experiments for comparing and applying upper thermal limits is an ongoing debate (Desforges et al., 2023). Our results show a large degree of difference in thermal maxima resulting from the selection of experimental design, in terms of absolute limits as well as the influence of prior temperature exposure. The logistic appeal of CT_max_ experiments is that they are fast to run and well suited for multiple comparisons. However, ecological relevance of rapid ramp rates is likely limited to extreme environments such as hydrothermal systems, tidepools and locations of anthropogenic thermal pollution (Bates and Morley, 2020). CT_max_ experiments are also suggested to be relevant to testing responses to heatwaves (Åsheim et al., 2020; Desforges et al., 2023; Ern et al., 2023), which may be true in certain regions or as climate change progresses. However, during the most extreme heatwave ever recorded in BC (summer of 2021 with air temperatures reaching 49.6°C), the maximum stream temperatures reached across hydrometric stations were 25.9–27.5°C with temperature ramp rates of 0.3–0.4°C h^−1^ on the day of peak temperature (calculated from raw data from three stations of 23 analysed in Callahan and Moore, 2025). This is still far below the rates of increase used in CT_max_ experiments, and our CT_max_ results would suggest that juvenile coho could have survived those temperatures. Conversely, our ITD_max_ results from the present study and Iacarella et al. (2024) indicate that mortality may have occurred. Conducting experiments that are more closely aligned with current and potential heatwave dynamics would be helpful for understanding and predicting mortality events during extreme conditions.

An organism's sensitivity to temperature change is also positively related to thermal maxima, such that individuals with high thermal maxima during acute exposure are unable to sustain tolerance over longer durations (Rezende et al., 2014). Likewise, Åsheim et al. (2020) and Bartlett et al. (2022) found weak to no relationships, respectively, comparing thermal maxima of the same individuals in rapid versus slow ramping trials. This further substantiates that CT_max_ results do not well represent responses to longer-term exposure that is more likely in the environment (Rezende et al., 2014; Bates and Morley, 2020). Others have identified that stochastic effects increase the probability of loss before reaching upper thermal limits with longer exposure duration, which could artificially reduce the estimation of thermal maxima with slower ramping (Santos et al., 2011). We found increased variation in the timing of LOE with our slower trials compared with previous ITD_max_ experiments (Iacarella et al., 2024). However, the variation in LOE of individuals could also signify real physiological differences that may be important in enabling population adaptation. Stochastic or otherwise, this individual variation based on physiological condition or genotype is likely more representative of how loss across a population would occur in the environment. Advocates of the rapid ramping design also point to confounding effects of resource depletion during longer experiments such as increased metabolic costs and starvation when food is withheld (Rezende et al., 2011; Santos et al., 2011). In our case, fish were fed to satiation throughout ITD_max_ experiments and other physiological requirements were met (e.g. flow-through water, dissolved oxygen).

Diel variation is also a fundamental aspect of most natural thermal regimes and has an important influence on physiological responses that is rarely captured in experimental designs (Morash et al., 2021). Morash et al. (2018) show that Atlantic salmon (*Salmo salar*) have reduced metabolic rates and aerobic scope, and increased stress responses when exposed to diel variation compared with static temperatures; this indicates potential physiological mechanisms contributing to our finding of reduced thermal maxima in the ITD_max_ experiments versus CT_max_ experiments. We recognize the benefits of CT_max_ experiments for logistical ease and to minimize variance in time of physiological failure between individuals for comparative purposes. However, there is significant value in conducting upper thermal limit experiments that aim to represent how organisms will fair under warming climates as this captures more realistic absolute limits and reduces the risk of inflated expectations of thermal maxima. Overestimating thermal maxima could have serious consequences for populations through the delay of mitigation actions, such as protection of thermal regimes or restoration activities. Characterizing future warming rates and diel variation will be important for guiding experimental designs that can improve our representation and understanding of potential organismal responses to climate change.

Population-level local adaptation of upper thermal limits has been presented as an important aspect of understanding species' responses and adaptability to warming (Bennett et al., 2019; McKenzie et al., 2021). In particular, local adaptation of thermal tolerance has been suggested to be particularly strong for populations with little gene flow or limited dispersal capacity, such as salmonids or some intertidal invertebrates (Somero, 2010; McKenzie et al., 2021). We found no clear evidence of local adaptation in upper thermal limits for juvenile coho salmon, which was corroborated by both experimental approaches. Though some comparisons between populations were statistically significant, they represented negligible differences in thermal maxima and no consistent pattern across trials. Studies on population differences in thermal maxima of other fish species have further yielded mixed results (Elliott and Elliott, 2010). CT_max_ comparisons of juvenile Chinook salmon (*Oncorhynchus tshawytscha*), juvenile brook trout (*Salvelinus fontinalis*) and adult common killifish (*Fundulus heteroclitus*) found differences in thermal maxima when comparing populations across the species' distributions, though results were influenced by body size differences among populations (Fangue et al., 2006; Stitt et al., 2014; Zillig et al., 2023). Conversely, differences in thermal maxima of juvenile sockeye salmon (*Oncorhynchus nerka*) populations were negated when body size was controlled (Chen et al., 2013), and no differences were found between juvenile rainbow trout (*Oncorhynchus mykiss*) populations with relatively similar body sizes (Myrick and Cech, 2000). From these few case studies, body mass appears to be a common confounding factor that influences interpretation of population-level local adaptation of upper thermal limits. Studies also found that medaka fish (*Oryzias latipes*) had different CT_max_ thermal limits after 7 years of rearing at different temperatures, but reverted back to the same thermal limit after one generation of rearing in common conditions, revealing a lack of genetic adaptation (Morla et al., 2025; Vallin et al., 2025); interestingly, body size differences created by hot and cold treatments were maintained after rearing under common conditions (Vallin et al., 2025). For anadromous fishes in particular, as indicated by studies on sockeye salmon, early juvenile life stages have shown similarity in upper thermal limits across populations, whereas adults influenced by disparate migration distances and spawning demands can have physiological and phenotypic differences that affect upper thermal limits (Eliason et al., 2011; Mayer et al., 2024). Overall, support for local adaptation of upper thermal limits as measured by thermal maxima experiments appears to be weaker than purported, at least for freshwater and anadromous fishes, and based on current study design limitations (Somero, 2010; Bennett et al., 2019; McKenzie et al., 2021; Mayer et al., 2024). Across taxa, few experiments that have found population differences in upper thermal limits have been able to distinguish plasticity or maternal effects from genetic adaptation, except primarily for *Drosophila* studies (Somero, 2010). The presence of local adaptation of upper thermal limits may be species and life-stage specific, and more rigorous experimentation is required where it is suspected. For taxa found to have limited or no local adaptation of upper thermal limits, use of species-level upper thermal limits to understand biogeographic patterns and predict sensitivity to climate change may be sufficient.

The degree of plasticity in upper thermal limits that is inferred from acute thermal maxima experimental designs may not necessarily lead to adaptation that is conserved over generations, as would be indicated by population-level local adaptation. Even plasticity as measured by responses to CT_max_ experimental designs (i.e. the slope of thermal maxima across acclimation temperatures) has shown evolutionary constraint in upper thermal limits across clades globally based on a finding of low plasticity relative to projected warming temperatures (Gunderson and Stillman, 2015). The hard ceiling in adaptability of upper thermal limits (Bennett et al., 2019; Morgan et al., 2020; McKenzie et al., 2021; Nati et al., 2021; Ern et al., 2023) further supports the relative importance of adaptative processes acting on behavioural shifts in thermoregulation rather than on absolute upper thermal limits (Gunderson and Stillman, 2015). Our ITD_max_ experimental design provides a realistic measure of those limits that can be applied more broadly across environments than CT_max_ results to advance assessments of where and when organisms may be most challenged by long-term warming.

## Supplementary Material

10.1242/jexbio.250748_sup1 Supplementary information
