# Supplementary material for: Addressing issues of experimental design, ecological realism and local adaptation for applications of ectotherm upper thermal limits
Source: J Exp Biol. 2025 Dec 2;228(23):jeb250748. doi: 10.1242/jeb.250748 (PMC12745932; doi:10.1242/jeb.250748)
Supplement: Supplementary information [file jexbio-228-250748-s1.pdf]

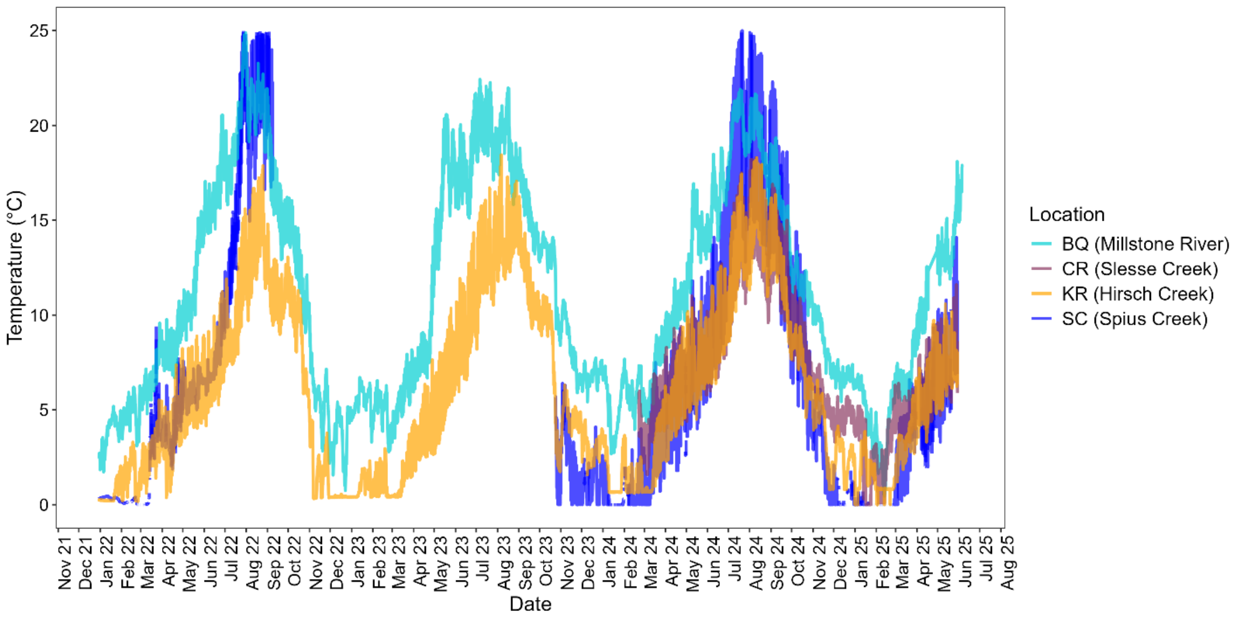

Fig. S1. Temperature profiles from hydrometric stations in the area of coho salmon population locations in BC. Hydrometric stations are Millstone River at Nanaimo, Slesse Creek near Vedder Crossing, Hirsch Creek near the mouth, Spius Creek near Canford.

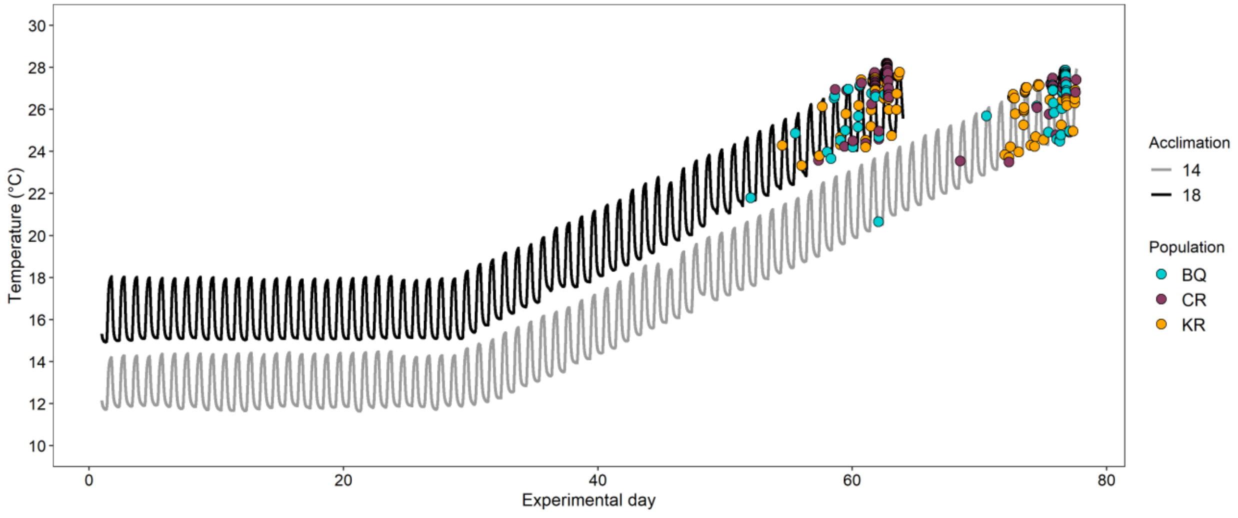

Fig. S2. Temperature profiles of the ITDmax experiment (lines) for 14 and 18 °C acclimation treatments, and juvenile coho salmon loss of equilibrium events (points) for populations from Big Qualicum (BQ), Chilliwack River (CR), and Kitimat River (KR). Experimental day starts at the beginning of acclimation, with temperature ramping for the trials beginning on day 29.

Journal of Experimental Biology • Supplementary information

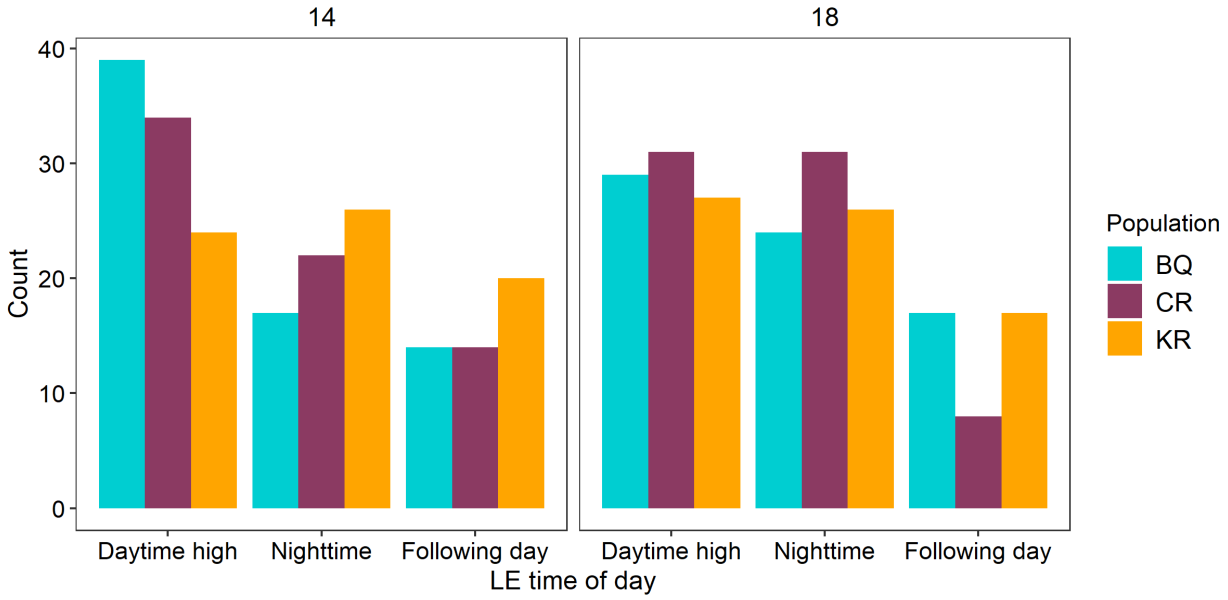

Fig. S3. Time of day when loss of equilibrium (LOE) occurred in 14 and 18 °C acclimation treatments for coho salmon populations from Big Qualicum (BQ), Chilliwack River (CR), and Kitimat River (KR). LOE occurred during a daytime high from 3-7pm, nighttime from 7pm-9am, or the following day from 9am-3pm. Counts are across two tank replicates per treatment.

Table S1. CTmax trial order across two acclimation temperatures, three populations ((Big Qualicum, ‘BQ’; Chilliwack River, ‘CR’; Kitimat River, ‘KR’; Spius Creek, ‘SC’), and two replicate tanks each.

**Time of day**	**Day 1**	**Day 2**	**Day 3**	**Day 4**
Morning	18 °C, CR, Rep 1	14 °C, KR, Rep 2	18 °C, CR, Rep 2	14 °C, BQ, Rep 1
Mid-day	18 °C, KR, Rep 1	14 °C, BQ, Rep 2	18 °C, BQ, Rep 2	14 °C, CR, Rep 1
Afternoon	18 °C, BQ, Rep 1	14 °C, CR, Rep 2	18 °C, KR, Rep 2	14 °C, KR, Rep 1

Journal of Experimental Biology • Supplementary information
